# Efficacy and safety of subcutaneous tocilizumab in rheumatoid arthritis over 1 year: a UK real-world, open-label study

**DOI:** 10.1093/rap/rkz010

**Published:** 2019-04-19

**Authors:** John D Isaacs, Abdelrazig Salih, Thomas Sheeran, Yusuf I Patel, Karen Douglas, Neil D McKay, Barbara Naisbett-Groet, Ernest Choy

**Affiliations:** 1 Arthritis Research UK Experimental Arthritis Treatment Centre, Institute of Cellular Medicine, Newcastle University, Newcastle-upon-Tyne; 2 Musculoskeletal Unit, Newcastle-upon-Tyne Hospitals NHS Foundation Trust, Newcastle-upon-Tyne; 3 Warrington and Halton Hospitals NHS Foundation Trust, Warrington Hospital, Warrington; 4 Rheumatology Centre, Cannock Chase Hospital, Cannock; 5 Department of Rheumatology, Hull Royal Infirmary, Hull; 6 Department of Rheumatology, Dudley Group NHS Foundation Trust, Dudley; 7 Rheumatic Diseases Unit, Western General Hospital, Edinburgh; 8 Roche Products Ltd, Welwyn Garden City; 9 CREATE Centre, Division of Infection and Immunity, Cardiff University, Cardiff, UK

**Keywords:** rheumatoid arthritis, tocilizumab, conventional synthetic DMARDs, subcutaneous, biological therapies, real world, United Kingdom

## Abstract

**Objective:**

The ACT-MOVE study assessed the real-world efficacy and safety of s.c. tocilizumab (TCZ-SC), provided as monotherapy or in combination with conventional synthetic DMARDs (csDMARDs) over 1 year, in patients with RA and an inadequate response to csDMARD therapy and/or first TNF inhibitor.

**Methods:**

In this UK multicentre, open-label phase IIIb study, patients received TCZ-SC 162 mg once weekly for 52 weeks as monotherapy or with csDMARDs. Efficacy and safety were evaluated at baseline, weeks 2 and 4 and every 4 weeks thereafter up to week 52.

**Results:**

Of 161 patients who received at least one dose of TCZ-SC, 21 (13.0%) received TCZ-SC alone and 140 (87.0%) TCZ-SC with a csDMARD(s). From baseline to week 52, there was a mean decrease in DAS28-ESR score among all patients (−3.68), and within monotherapy (−3.75) and combination therapy (−3.67) groups. The proportion of patients who achieved DAS28 clinical remission (DAS28-ESR <2.6) at week 52 was 75.4% (95% CI 66.8, 82.8). At the same time point, ≥80% of patients who remained on TCZ-SC achieved DAS28 clinical remission or had low disease activity (DAS28-ESR ≥2.6 and ≤3.2). Overall, 6.2% of patients had at least one serious adverse event (10.2/100 patient-years), and there was one death; 11.2% of patients discontinued owing to adverse events.

**Conclusion:**

TCZ-SC was effective and tolerated in a real-world setting over 1 year. The efficacy of TCZ-SC was similar whether given as monotherapy or with csDMARDs; its safety profile was consistent with that previously established.

**Trial registration:**

ClinicalTrials.gov, http://www.clinicaltrials.gov, NCT02046603.

**Key messages**

- 75% of ‘real-world’ RA patients failing conventional synthetic DMARDs/first TNF inhibitor achieve remission with s.c. tocilizumab.
- The efficacy of s.c. tocilizumab in RA was similar as monotherapy or combined with conventional synthetic DMARDs.
- The real-world safety profile of s.c. tocilizumab in RA was consistent with that previously established.

## Introduction

RA is an autoimmune disease affecting ∼1% of the global population that is associated with painful inflammation and destruction of the joints and surrounding tissue [1]. In Europe, recommended treatment involves early initiation of conventional synthetic DMARDs (csDMARDs) and, if an adequate response is not achieved, addition of a biological therapy, such as tocilizumab (TCZ), abatacept or a TNF inhibitor, or a targeted synthetic DMARD (i.e. a Janus kinase inhibitor) [2].

TCZ is a recombinant, humanized, monoclonal antibody targeting soluble and membrane-bound IL-6 receptors. IL-6 is a pro-inflammatory cytokine with a role in several inflammatory diseases, including RA [3, 4]. TCZ was initially approved as an i.v. formulation (TCZ-IV) for treatment of patients with moderate-to-severe active RA and an inadequate response or intolerance to previous csDMARDs or anti-TNF therapy. Data from several phase III trials demonstrated the efficacy and safety of TCZ-IV alone and with csDMARD therapy in this patient population [5–9]. Longer-term data from the LITHE trial also showed that the efficacy and safety of TCZ-IV were maintained for up to 5 years [10].

An s.c. formulation of TCZ (TCZ-SC) was later developed and approved [11, 12]. Many RA patients express a preference for s.c. administration, preferring the convenience of home self-administration with ready-to-use prefilled syringes [13]. The efficacy and safety of TCZ-SC as monotherapy or in combination with csDMARD therapy was evaluated in three phase III studies in patients with RA and an inadequate response to csDMARDs [14–16]. Data from the SUMMACTA study showed non-inferiority of TCZ-SC (162 mg weekly) to TCZ-IV (8 mg/kg every 4 weeks), both in combination with csDMARDs, with regard to the proportion of patients achieving an ACR 20% improvement (ACR20) response at week 24 [14]. In the BREVACTA study, ACR20 at 24 weeks with TCZ-SC (162 mg every 2 weeks) plus csDMARDs was superior to s.c. placebo with csDMARDs [15]. In the MUSASHI study, TCZ-SC monotherapy (162 mg every 2 weeks) was shown to be non-inferior to TCZ-IV monotherapy (8 mg/kg every 4 weeks) with respect to ACR20 at week 24 [16]. In all these studies, TCZ-SC was well tolerated, with a safety profile consistent with TCZ-IV [14–16]. Long-term extension studies with TCZ-SC, ranging from 84 to 108 weeks’ duration, showed durable efficacy and maintenance of a favourable safety profile [17–19].

Recently, ‘real-world’ use of TCZ-SC has been evaluated prospectively, with the intention of providing more information about outcomes when treatment is administered at home. TOZURA is a multinational, open-label, phase IV study programme designed to evaluate the efficacy and safety of TCZ-SC as monotherapy and in combination with csDMARD(s) in adult patients with moderate-to-severe RA across a broad geographical setting. Using a common design framework, the programme comprises 11 studies in 22 countries [20]. Recently published data from the overall TOZURA programme confirmed the existing efficacy and safety profiles of TCZ-SC, showing comparable results when it was used alone or in combination over 24 weeks [20].

The UK-based ACT-MOVE study was part of the TOZURA programme and was designed to assess the real-world efficacy of TCZ-SC, as monotherapy or in combination with csDMARD therapy, for up to 1 year in RA patients with an inadequate response to csDMARDs and/or first TNF inhibitor.

## Methods

### Patients

The study population included TCZ-naïve adults (≥18 years old) with active RA, according to the revised 1987 ACR criteria or 2010 EULAR/ACR criteria, and an inadequate response to current csDMARD therapy or first TNF inhibitor. Past TNF inhibitors may have been given as monotherapy or in combination with MTX or another csDMARD. An inadequate response to anti-TNF treatment was defined as a DAS using 28 joints (DAS28) improvement of <1.2, or patients not achieving a DAS28 of ≤3.2 according to a treat-to-target strategy. Inadequate response to csDMARD therapy was assessed according to local guidelines.

Key exclusion criteria included major surgery ≤8 weeks before screening or planned major surgery ≤6 months after baseline, on-going rheumatic autoimmune disease other than RA, functional class IV status, prior history of or current inflammatory joint disease other than RA, treatment with any investigational agent ≤4 weeks or ≤5 half-lives of the investigational agent before screening (whichever was longer), IA or parenteral glucocorticoids or immunization with a live/attenuated vaccine ≤4 weeks before screening, treatment with any cell-depleting therapies or alkylating agents, and treatment with i.v. γ-globulin or plasmapheresis ≤6 months before baseline. Patients were also excluded if they had evidence of serious uncontrolled concomitant disease, a history of diverticulitis or symptomatic lower gastrointestinal (GI) conditions that might predispose to perforation, any active infections, positive hepatitis B surface antigen or hepatitis C antibody, history of or currently active malignancy, or serious allergies to biological agents. Complete eligibility criteria are provided in supplementary Table S1, available at *Rheumatology Advances in Practice* online.

Final protocols, amendments and informed consent documentation were approved by the local institutional review boards or independent ethics committees of the study centres. All patients provided written, informed consent, according to the *Declaration of Helsinki*.

### Study design

ACT-MOVE (NCT02046603) was a real-world, multicentre, open-label, single-arm, phase IIIb trial performed between March 2014 and August 2016. Patients received TCZ 162 mg once a week for 52 weeks, administered by s.c. injection as a single, fixed dose irrespective of body weight. Each TCZ dose was supplied in a 1-ml, ready-to-use, single-use, prefilled syringe. Concomitant treatment with csDMARDs, including AZA, chloroquine, HCQ, LEF, MTX or SSZ, was permitted if the patient had maintained a stable dose for ≥4 weeks before baseline assessment. Concomitant csDMARDs could be used alone or in combination, except for the combination of MTX and LEF. Oral NSAIDs and glucocorticoids (≤10 mg/day prednisone or equivalent) were permitted if patients maintained a stable dose for ≥4 weeks before baseline.

After administration of the first s.c. injection under close investigator supervision, patients or caregivers could administer subsequent doses of TCZ-SC at home. Recommended injection sites were the front of the middle thighs and the lower part of the abdomen below the navel, except for the two-inch area directly around the navel. The outer area of the upper arms could also be used by caregivers administering an injection.

### Objectives and assessments

The primary study objective was to assess the efficacy of TCZ-SC (as monotherapy or in combination) in patients with an inadequate response to csDMARDs and/or first TNF inhibitor. Secondary objectives included evaluating TCZ-SC safety and tolerability, efficacy over time, the proportion of patients who achieved low disease activity, the proportion of patients who achieved remission, and adherence to MTX (in patients prescribed MTX in combination with TCZ-SC).

Efficacy and safety were evaluated at baseline, weeks 2 and 4 and every 4 weeks thereafter up to week 52, unless specified otherwise, with an additional follow-up safety evaluation performed 8 weeks after study completion. Efficacy assessments included change in DAS28 using ESR, change in clinical disease activity index (CDAI), change in simplified disease activity index (SDAI), ACR response scores, EULAR response, change in total swollen joint count of 28 joints and change in total tender joint count of 28 joints. Changes in serum CRP and ESR were also assessed. Patient-reported outcome assessments comprised global assessment of disease activity visual analog scale, RA-related pain visual analog scale, HAQ-disability index, functional assessment of chronic illness therapy – fatigue, and an MTX adherence questionnaire. The MTX adherence questionnaire, developed for this study, asked MTX-prescribed patients, ‘Over the last 3 months you were prescribed 12 doses of MTX, how many (approximately) have you taken?’.

Safety assessments included monitoring of adverse events [AEs; e.g. incidence and severity of treatment-emergent adverse events (TEAEs), incidence of treatment-emergent serious adverse events (TESAEs), TEAEs leading to study withdrawal or dose modification and TEAEs of special interest]. The TEAEs of special interest were identified via standardized Medical Dictionary for Regulatory Activities (MedDRA) query and included serious infections, GI perforations, demyelinating disorders, haematological abnormalities and bleeding events, hepatic enzyme elevation, cardiovascular disease and elevated lipids, malignancies, local injection site reactions and anaphylaxis/hypersensitivity reactions. Other safety variables included standard laboratory parameters, physical examination findings and vital signs. Samples for anti-drug antibody (ADA) testing were collected at baseline, weeks 12 and 24, at completion or early withdrawal visit and at the follow-up visit 8 weeks after the final dose.

### Statistical analyses

Study analyses were exploratory and primarily descriptive. No hypothesis testing was performed. A sample size of 160 patients was planned. Assuming an s.d. of 1.4 DAS28-ESR units and 13 CDAI units, respectively, the expected precision of the estimate, as measured by the 95% CI around the mean change from baseline, was 0.43 for DAS28-ESR and 4.0 for CDAI. For 80% power, the detectable change in the two variables, using a *t*-test at the 5% significance level, was 0.31 units for DAS28-ESR and 2.90 units for CDAI.

The proportion of patients who achieved DAS28 remission (DAS28-ESR <2.6) at weeks 24 and 52 was calculated along with 95% Clopper–Pearson CIs. Safety event incidence rates per 100 patient-years (PY) of TCZ-SC exposure were estimated using the Poisson distribution. The Clopper–Pearson method was used to calculate 95% CI for the incidence of TEAEs of special interest. Descriptive statistical methods were used to evaluate all other efficacy end-points and safety parameters. All reported analyses were performed in the full analysis set (FAS), which included all enrolled patients who received at least one dose of TCZ-SC.

## Results

### Patient disposition and baseline characteristics

Overall, 162 patients were enrolled across 38 UK centres; 131 and 31 patients had experienced an inadequate response to current csDMARD therapy or first TNF inhibitor, respectively. The FAS included 161 patients, because one patient in the monotherapy group did not receive TCZ-SC. Twenty-one FAS patients (13.0%) received TCZ-SC monotherapy, and 140 FAS patients (87.0%) received TCZ-SC in combination with csDMARDs. During the 52-week study, five patients (23.8%) discontinued from monotherapy and 33 patients (23.6%) from combination therapy; AEs were the most common reason for withdrawal (supplementary Fig. S1, available at *Rheumatology Advances in Practice* online).

Patient demographics and disease characteristics at baseline are shown in Table 1. The mean baseline DAS28-ESR score for all patients was 5.53. Demographics were generally comparable between monotherapy and combination therapy groups. However, glucocorticoid use was higher in the monotherapy group than in the combination group (33.3 and 21.4% of patients, respectively); in addition, more patients in the monotherapy group had received prior anti-TNF treatment (42.8 and 15.7%, respectively).

**Table 1:** Baseline patient demographics and disease characteristics (full analysis set)

Characteristic	TCZ-SC monotherapy (*n* = 21)	TCZ-SC + csDMARD (*n* = 140)	Total population (*n* = 161)
Age, mean (range), years	53.9 (27–79)	55.3 (32–81)	55.1 (27–81)
Female, *n* (%)	16 (76.2)	104 (74.3)	120 (74.5)
Race, *n* (%)			
White	20 (95.2)	135 (96.4)	155 (96.3)
Other	1 (4.8)	5 (3.6)	6 (3.7)
CRP, mean (s.d.), mg/l	15.4 (19.7)	16.1 (25.5)	16.0 (24.8)
RF-positive, *n* (%)	10 (47.6)	90 (64.3)	100 (62.1)
Anti-CCP_2_-positive, *n* (%)	12 (57.1)	101 (72.1)	113 (70.2)
Disease activity, mean (s.d.)			
DAS28-ESR	5.52 (1.01)	5.53 (1.26)	5.53 (1.23)
CDAI	29.69 (11.209)	30.88 (10.953)	30.73 (10.959)
SDAI	31.23 (11.892)	32.33 (11.620)	32.19 (11.624)
HAQ-DI	1.81 (0.56)	1.74 (0.64)	1.75 (0.63)
Concomitant RA medications, *n* (%)			
MTX	0 (0)	43 (30.7)^a^	43 (26.7)
Glucocorticoids	7 (33.3)	30 (21.4)	37 (23.0)
Prior anti-TNF treatment for RA, *n* (%)	9 (42.8)	22 (15.7)	31 (19.3)

a Data shown are baseline data; csDMARDs could be added after baseline in this group. CDAI: clinical disease activity index; csDMARD: conventional synthetic DMARD; DAS28: DAS for 28 joints; DI: disability index; SDAI: simplified disease activity index; TCZ-SC: tocilizumab s.c.

### Efficacy

**Fig. 1:**
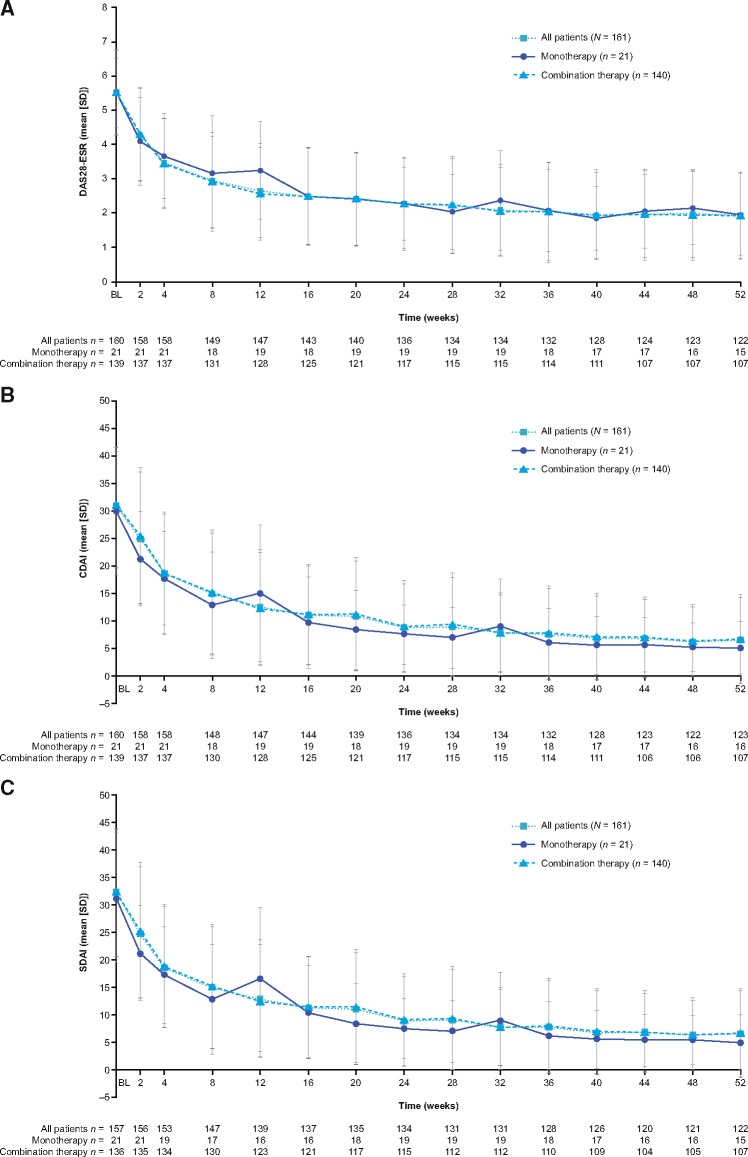
Mean DAS28-ESR (**A**), clinical disease activity index (**B**) and simplified disease activity index (**C**) scores over 52 weeks (full analysis set) BL: baseline; CDAI: clinical disease activity index; DAS28: DAS for 28 joints; SDAI: simplified disease activity index.

**Fig. 2:**
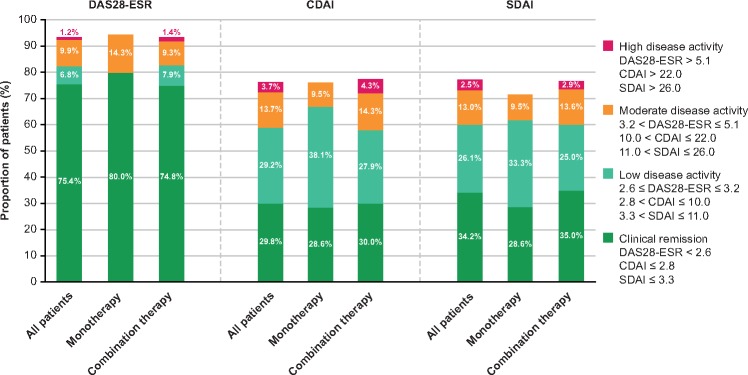
DAS28-ESR, clinical disease activity index and simplified disease activity index disease activity at week 52 (full analysis set) Data are shown as a percentage of the patients in each group at baseline (all patients, *n* = 161; monotherapy, *n* = 21; combination therapy, *n* = 140). As a result of patients withdrawing from the study, the percentages at week 52 do not total 100. CDAI: clinical disease activity index; DAS28: DAS for 28 joints; SDAI: simplified disease activity index.

There were mean decreases from baseline in DAS28-ESR scores at all time points (Fig. 1A). From baseline to week 52, the mean change in DAS28-ESR score for all patients was −3.68 (−3.75 and −3.67 in the monotherapy and combination groups, respectively). The proportion of all patients achieving DAS28 clinical remission (DAS28-ESR <2.6) was 66.2% (95% CI 57.6, 74.1) at week 24 and 75.4% (95% CI 66.8, 82.8) at week 52. The proportion of patients achieving DAS28 clinical remission at week 52 was >70% in both the monotherapy and combination groups (Fig. 2). At week 52, ≥80% of patients remaining on TCZ-SC therapy were assessed as achieving DAS28 clinical remission or having low disease activity (DAS28-ESR ≥2.6 and ≤3.2; Fig. 2).

At all time points, there was a mean decrease from baseline in CDAI scores (Fig. 1B). From baseline to week 52, the mean change in CDAI score was −24.55 (−25.48 and −24.42 in the monotherapy and combination groups, respectively). At week 52, the proportion of patients who achieved CDAI clinical remission (CDAI ≤2.8) was 29.8% in all patients and 28.6 and 30.0% in the monotherapy and combination groups, respectively (Fig. 2). The proportion of patients falling into other CDAI categories at week 52 was as follows: low disease activity (CDAI >2.8 to ≤10.0): 29.2, 38.1 and 27.9%; moderate disease activity (CDAI >10.0 to ≤22.0): 13.7, 9.5 and 14.3%; and high disease activity (CDAI >22.0): 3.7, 0.0 and 4.3% (Fig. 2). At all time points, there was a mean decrease from baseline in SDAI scores for all patients and in both groups (Fig. 1C). SDAI disease activity data at week 52 were similar to CDAI data (Fig. 2).

**Fig. 3:**
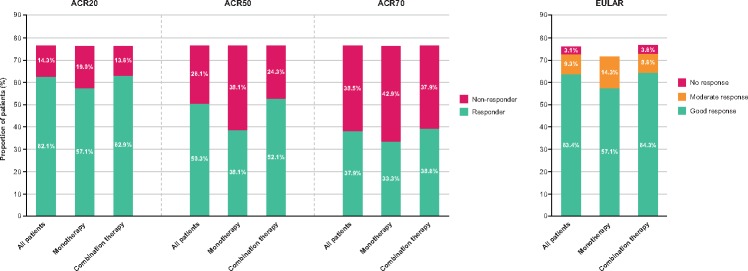
ACR and EULAR responses at week 52 (full analysis set) Data are shown as a percentage of the patients in each group at baseline (all patients, *n* = 161; monotherapy, *n* = 21; combination therapy, *n* = 140). As a result of patients withdrawing from the study, the percentages at week 52 do not total 100.

The proportion of all patients achieving ACR20, ACR50 and ACR70 responses increased over time (week 2 *vs* week 52: ACR20, 18.0 *vs* 62.1%; ACR50, 3.1 *vs* 50.3%; ACR70, 0.0 *vs* 37.9%). At week 52, 38.1% of patients in the monotherapy group and 52.1% in the combination group had achieved an ACR50 response (Fig. 3). The proportion of patients with a good EULAR response increased over time (19.9% at week 2 *vs* 63.4% at week 52 for all patients). At week 52, 57.1% of patients in the monotherapy group and 64.3% in the combination group achieved a good EULAR response (Fig. 3). There were decreases in swollen joint count of 28 joints and tender joint count of 28 joints of >75% from baseline to week 52 for all patients and in both groups (supplementary Tables S2 and S3, available at *Rheumatology Advances in Practice* online). Serum concentrations of CRP and ESR decreased at all time points (supplementary Figs S2 and S3, available at *Rheumatology Advances in Practice* online).

**Fig. 4:**
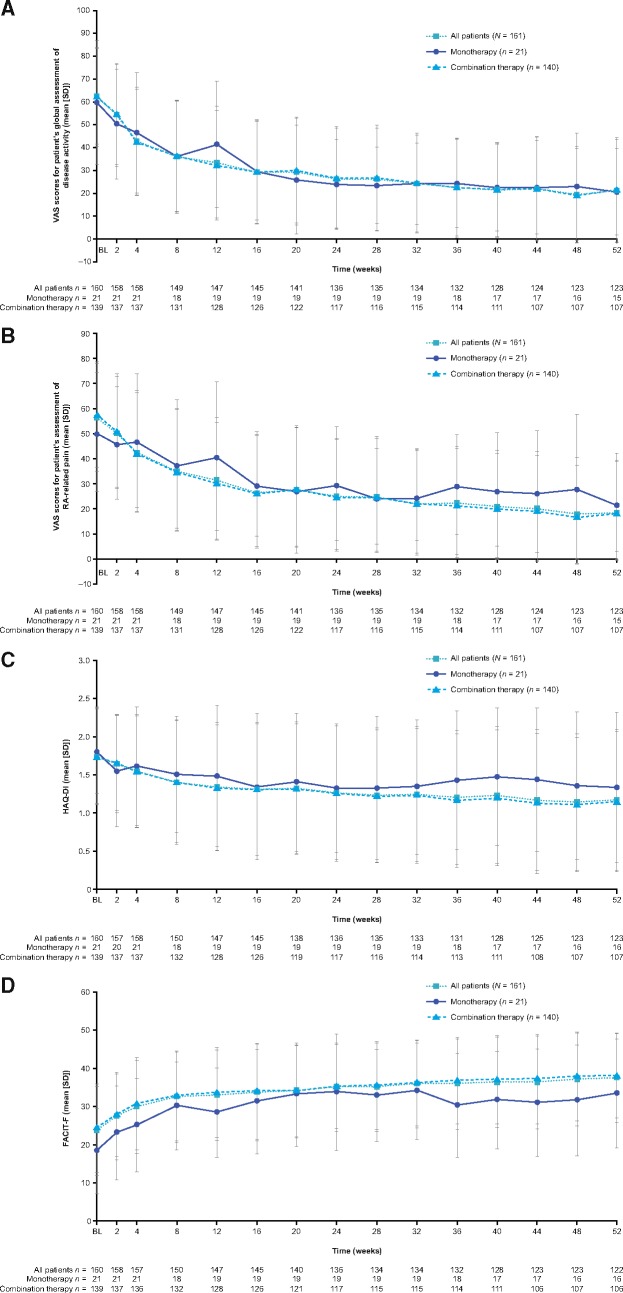
Mean change in select patient-reported outcome assessment scores over time (weeks 1–52; full analysis set) (**A**) VAS scores for patient global assessment of disease activity. (**B**) VAS score for patient assessment of RA-related pain. (**C**) HAQ-DI scores. (**D**) FACIT-F scores. BL: baseline; DI: disability index; FACIT-F: functional assessment of chronic illness therapy – fatigue; PRO: patient-reported outcome; VAS: visual analog scale.

For patient-reported outcome assessments, visual analog scale scores decreased for patient global assessment of disease activity and for RA-related pain at all time points (Fig. 4A and B). Mean baseline to week 52 changes for all patients, monotherapy and combination, respectively, were −40.8, −44.9 and −40.2 for disease activity, and −36.5, −33.4 and −36.9 for RA-related pain. There were decreases in HAQ-disability index scores from baseline to week 52 in all patients and both groups, indicating an improvement in disability (mean baseline to week 52 changes were −0.56, −0.47 and −0.57 for all patients, monotherapy and combination, respectively; Fig. 4C). The functional assessment of chronic illness therapy – fatigue score increased at all time points, indicating a decrease in fatigue (mean baseline to week 52 changes were 13.8, 16.9 and 13.3 for all patients, monotherapy and combination, respectively; Fig. 4D).

Of the 140 patients who received TCZ in combination with csDMARDs, 100 (71.4%) had previously been, or were currently, receiving MTX [although at baseline, MTX was listed as a concomitant RA medication in only 43 patients (30.7%; Table 1)]. Among the aforementioned 100 patients, 97 completed the MTX adherence questionnaire at baseline. The number of patients completing this questionnaire at weeks 12, 24, 36 and 52 was 80, 65, 60 and 60, respectively. Mean adherence to MTX in patients completing the questionnaire was >90% at all time points.

### Safety

Total mean TCZ-SC exposure was 321.6 and 310.4 days in the monotherapy and combination groups, respectively. A total of 157 patients (97.5%) had at least one TEAE, with a similar proportion of patients affected in the monotherapy and combination groups (95.2 *vs* 97.9%, respectively). The TEAE rate per 100 PY was 966.1 for all patients but was higher in the combination group than in the monotherapy group (997.7 *vs* 762.6). Most TEAEs were mild to moderate in severity (Table 2). One patient had a TEAE that was considered life-threatening (atrial fibrillation) and one patient died (pulmonary fibrosis); both patients were in the combination group.

**Table 2:** Summary of treatment-emergent adverse events (full analysis set)^a^

Events	TCZ-SC monotherapy (*n* = 21)	TCZ-SC + csDMARD (*n* = 140)	Total population (*n* = 161)
TEAEs			
Total number of TEAEs	141	1187	1328
Patients with at least one TEAE, *n* (%)	20 (95.2)	137 (97.9)	157 (97.5)
Most extreme TEAE intensity, *n* (%)			
Mild	6 (28.6)	48 (34.3)	54 (33.5)
Moderate	8 (38.1)	66 (47.1)	74 (46.0)
Severe	6 (28.6)	21 (15.0)	27 (16.8)
Life-threatening	0 (0.0)	1 (0.7)	1 (0.6)
Rate, per 100 PY of exposure	762.6	997.7	966.1
Treatment-related TEAEs			
Patients with at least one treatment-related TEAE, *n* (%)	9 (42.9)	103 (73.6)	112 (69.6)
Rate, per 100 PY of exposure	227.2	290.0	281.5
TEAEs leading to discontinuation			
Patients with at least one TEAE leading to discontinuation, *n* (%)	3 (14.3)	15 (10.7)	18 (11.2)
Rate, per 100 PY of exposure	21.6	16.8	17.5
TESAEs			
Patients with at least one TESAE, *n* (%)	3 (14.3)	7 (5.0)	10 (6.2)
Rate, per 100 PY of exposure	32.5	6.7	10.2
Serious infections			
Patients with at least one event, *n* (%)	1 (4.8)	3 (2.1)	4 (2.5)
Rate, per 100 PY of exposure	10.8	2.5	3.6
TEAEs of special interest^b^			
Patients with at least one TEAE of special interest, *n* (%)	9 (42.9)	61 (43.6)	70 (43.5)
	[95% CI 27.8, 66.0]	[95% CI 35.2, 52.2]	[95% CI 35.7, 51.5]
Deaths, *n* (%)	0 (0.0)	1 (0.7)^c^	1 (0.6)^c^

a If a patient experienced more than one TEAE, the patient was counted once at the most intense or most related event.

b Identified via SMQ.

c Owing to chronic pulmonary fibrosis. csDMARD: conventional synthetic DMARD; PY: patient-years; SMQ: standardized Medical Dictionary for Regulatory Activities (MedDRA) query; TCZ-SC: tocilizumab s.c.; TEAE: treatment-emergent adverse event; TESAE: treatment-emergent serious adverse event.

The most common class of TEAEs was infections, reported by 70.2% of all patients (76.2 and 69.3% in the monotherapy and combination groups, respectively). Nasopharyngitis was the most frequently reported individual TEAE (23.6% of all patients; supplementary Table S4, available at *Rheumatology Advances in Practice* online). TEAEs resulting from injection site reactions were experienced by 34 (21.1%) patients [four (19.0%) and 30 (21.4%) in the monotherapy and combination groups, respectively]. No patient had a GI perforation.

TEAEs considered by the investigator to be related to study medication were reported in 112 patients (69.6%); the proportion of patients with these events was higher in the combination group than in the monotherapy group (Table 2). Fourteen TESAEs were reported in 10 patients (6.2%; supplementary Table S5, available at *Rheumatology Advances in Practice* online), with a rate of 10.2 per 100 PY; four patients (2.5%) experienced a serious infection (3.6 per 100 PY; Table 2). Eighteen patients (11.2%) discontinued study treatment owing to TEAEs; rates of discontinuation in the monotherapy and combination groups were broadly similar (Table 2). The most common classes of TEAEs causing withdrawal were infections (*n* = 4) and GI disorders, abnormal laboratory investigations and skin/s.c. tissue disorders (*n* = 3 each). Nineteen patients (11.8%; three in the monotherapy group and 16 in the combination group) experienced a total of 25 TEAEs that led to TCZ-SC dose modification (dosing interrupted and/or dose frequency reduced).

Seventy patients (43.5%; 95% CI 35.7, 51.5) had a TEAE of special interest (Table 2). TEAEs of special interest that occurred in ≥3% of patients in any group are shown in supplementary Table S6 (available at *Rheumatology Advances in Practice* online); the most common were rash, injection site bruising and contusion.

At week 24, 2 of 135 patients (1.2%) had an ADA-positive result; both patients were in the combination group, and one had neutralizing antibodies to TCZ-SC. Samples were collected from 24 patients at the 8-week follow-up visit; none was ADA positive at this time point.

## Discussion

ACT-MOVE was a UK real-world study performed within the TOZURA phase IV common-framework programme. The primary objective was to assess the efficacy of TCZ-SC, as monotherapy or in combination with csDMARD(s), in patients with an inadequate response to current csDMARD therapy or first TNF inhibitor, up to week 52. Study findings showed that in a population of patients with high mean disease activity at baseline, TCZ-SC given alone or in combination with csDMARDs led to mean decreases from baseline in DAS28-ESR scores and an increase in the proportion of patients in DAS28 clinical remission over the treatment period. Other efficacy end-points, including CDAI, SDAI, ACR response scores, EULAR response, tender joint count of 28 joints, swollen joint count of 28 joints and patient-reported outcomes, also showed an improvement from baseline over 52 weeks with TCZ-SC alone or in combination. TCZ-SC was generally well tolerated as monotherapy and in combination, with safety data consistent with the existing profile of TCZ-SC [14–16].

Outcomes in ACT-MOVE were generally comparable to those from phase III trials of TCZ-IV, acknowledging differences in patient populations and trial designs [5–9]. In a recent study of TCZ-IV plus MTX in patients with an inadequate response to DMARDs, tapering MTX was non-inferior to continuing stable MTX in terms of maintaining a EULAR response in patients with a good/moderate EULAR response at week 24. As observed in ACT-MOVE, TCZ treatment was well tolerated and efficacious in combination with csDMARDs [21]. Efficacy of TCZ treatment in ACT-MOVE was similar whether as monotherapy or with csDMARDs, concurring with a meta-analysis of randomized controlled trials that compared these two approaches [22]. ACT-MOVE efficacy data were also comparable to those from the overall TOZURA programme and other phase III trials of TCZ-SC [14–16, 20], although the proportion of patients achieving DAS28 clinical remission at week 24 was higher in ACT-MOVE than in TOZURA for monotherapy (73.7% ACT-MOVE, 59.0% TOZURA) and higher than in the SUMMACTA study for combination therapy (65.0% ACT-MOVE, 38.0% SUMMACTA) [14, 20]. ACR20 response rate, the primary end-point in SUMMACTA, however, was comparable (71.4% ACT-MOVE, 69.4% SUMMACTA).

In terms of safety, the rate of TEAEs in ACT-MOVE per 100 PY was higher than the rate of AEs in the overall TOZURA programme (966.1 ACT-MOVE, 622.4 TOZURA), although the rate of TESAEs per 100 PY was lower (10.2) than the rate of serious AEs in TOZURA (14.6). In both studies, infections were the most commonly reported events; the rate of serious infections per 100 PY was 3.6 in both ACT-MOVE and TOZURA. With reference to other specific TEAEs, cross-study comparisons across ACT-MOVE and TOZURA are difficult, because their frequency was reported as the number (proportion) of patients affected, and the two studies were of different durations (24 and 54 weeks, respectively). It is possible that the apparently higher rate of non-serious AEs in ACT-MOVE might be related partly to differences in study treatment; specifically, that a slightly higher proportion of patients in this study received combination treatment with TCZ-SC and a csDMARD *vs* TCZ-SC monotherapy (ACT-MOVE: 87 *vs* 13%; TOZURA: 80 *vs* 20%). Any increase in AEs reported here *vs* TOZURA is unlikely to reflect a need for extra safety monitoring given that it appears related to non-serious events only. Of note, the per 100 PY rate of TESAEs in ACT-MOVE was lower than that of serious AEs in a close to clinical practice study of TCZ-IV as monotherapy or in combination with DMARDs (10.2 *vs* 20.1) [23]. Further comparisons with that TCZ-IV study are, however, compromised by differences in study length and AE data presentation. Overall, the ACT-MOVE safety data reported here are generally reassuring given that its real-world setting means patients are likely to have more co-morbidities than those enrolled in clinical trials. Co-morbidities were reported in 36% of ACT-MOVE patients at baseline [most commonly, hypertension (21%), hyperlipidaemia (8%), type 2 diabetes (4%) and osteoporosis (4%)].

It is well established that biological therapies can elicit the production of ADAs, which may negatively impact treatment through reduced exposure/efficacy and triggering of hypersensitivity reactions [24]. Consistent with overall data from TOZURA [20], immunogenicity in ACT-MOVE was infrequent in both monotherapy and combination groups.

The present findings from ACT-MOVE are similar to those from a Spanish, retrospective, observational study of switching from TCZ-IV to TCZ-SC in RA (DAS28 clinical remission rates in that study were 75.5 and 87.3% at weeks 24 and 52, respectively) [25]. Data from these studies, alongside findings of TOZURA, provide a growing body of evidence showing that the efficacy and safety of TCZ-SC in the real world are similar to observations during clinical development. However, the small number of patients enrolled onto the TCZ-SC monotherapy arm of ACT-MOVE limits the conclusions that can be drawn specifically from this group. This small sample size might account for the potential imbalance at baseline between monotherapy and combination groups in terms of glucocorticoid use and prior anti-TNF treatment. Furthermore, the present study, and the overall TOZURA programme, lacked a control arm and are subject to the limitations generally associated with real-world studies, such as inclusion and expectation bias. These limitations, however, are at least partly offset by the benefits of including a broader, less selected patient population. A novel aspect of our study *vs* the overall TOZURA programme was assessment of MTX adherence in patients prescribed MTX in combination with TCZ-SC. At all time points, MTX adherence was >90%, although the number of patients completing the adherence questionnaire was relatively low at post-baseline assessments.

In summary, findings from ACT-MOVE demonstrate the efficacy of TCZ-SC in patients with RA in a real-world setting over a 1-year period. The efficacy of TCZ-SC was similar whether it was prescribed as monotherapy or in combination with csDMARDs. The safety profile was consistent with that previously established for TCZ-SC.

## Supplementary Material

Supplementary Data
