# Supplementary material for: Efficacy and safety of subcutaneous tocilizumab in rheumatoid arthritis over 1 year: a UK real-world, open-label study
Source: Rheumatol Adv Pract. 2019 Apr 19;3(1):rkz010. doi: 10.1093/rap/rkz010 (PMC6649953; doi:10.1093/rap/rkz010)
Supplement: Supplementary Data [file rkz010_supp.docx]

**Supplementary material**

**Supplementary Table S1.** Full inclusion and exclusion criteria

**Inclusion criteria**

Patients must meet the following criteria for study entry:

- Able and willing to give written, informed consent and comply with the requirements of the study protocol.
- Patients at least 18 years of age.
- Patients with a diagnosis of active rheumatoid arthritis (RA) according to the revised (1987) American College of Rheumatology (ACR) criteria or European League Against Rheumatism (EULAR)/ACR (2010) criteria.
- Oral glucocorticoids (≤10 mg/day prednisone or equivalent) and non-steroidal anti-inflammatory drugs (NSAIDs; up to the maximum recommended dose) are permitted if on a stable dose regimen for ≥4 weeks prior to baseline.
- Permitted non-biologic disease-modifying anti-rheumatic drugs (DMARDs) are allowed if at a stable dose for at least 4 weeks prior to baseline.
- Receiving treatment on an outpatient basis, not including tocilizumab.
- Females of childbearing potential and males with female partners of childbearing potential may participate in this study only if using a reliable means of contraception (e.g. physical barrier [patient or partner], contraceptive pill or patch, spermicide and barrier, or intrauterine device) during the study. Females of childbearing potential and males with female partners of childbearing potential must use a reliable means of contraception for at least 3 months following the last dose of tocilizumab.
- If female of childbearing potential, the patient must have a negative pregnancy test at screening and baseline visits.
- Patients who have an inadequate response to current non-biologic DMARD therapy or the first anti-tumour necrosis factor (TNF) agent (in monotherapy or in combination with methotrexate [MTX] or other non-biologic DMARDs). Inadequate response to anti-TNF treatment is defined by the National Institute for Health and Care Excellence (NICE) as a disease activity score using 28 joints (DAS28) improvement of less than 1.2 or patients achieving a 1.2 reduction in DAS28 but not achieving low disease activity (current DAS28-erythrocyte sedimentation rate [ESR] above 3.2) according to a treat-to-target strategy and have not been previously exposed to treatment with tocilizumab. Inadequate response to non-biologic DMARD therapy will be assessed according to local guidelines and the patients will need to be eligible for biologic therapy according to local guidelines.

**Exclusion criteria**

General:

- Major surgery (including joint surgery) within 8 weeks prior to screening or planned major surgery within 6 months following baseline.
- Rheumatic autoimmune disease other than RA, including systemic lupus erythematosus, mixed connective tissue disorder, scleroderma, polymyositis, or significant systemic involvement secondary to RA (e.g. vasculitis, pulmonary fibrosis or Felty’s syndrome). Secondary Sjögren’s syndrome with RA is permitted.
- Functional Class IV as defined by the ACR Classification of Functional Status in Rheumatoid Arthritis.
- Diagnosis of juvenile idiopathic arthritis or juvenile RA, and/or RA before the age of 16 years.
- Prior history of or current inflammatory joint disease other than RA (e.g. gout, Lyme disease, seronegative spondyloarthropathy including reactive arthritis, psoriatic arthritis and arthropathy of inflammatory bowel disease).

Excluded previous or concomitant therapy:

- Exposure to tocilizumab (either intravenous [IV] or subcutaneous [SC]) at any time prior to baseline.
- Treatment with any investigational agent within 4 weeks (or five half-lives of the investigational drug, whichever is longer) of screening.
- Previous treatment with any cell-depleting therapies, including investigational agents or approved therapies, some examples are alemtuzumab, anti-CD4, anti-CD5, anti-CD3, anti-CD19 and anti-CD20.
- Treatment with IV gamma globulin, plasmapheresis within 6 months of baseline.
- Intra-articular or parenteral glucocorticoids within 4 weeks prior to baseline.
- Immunisation with a live/attenuated vaccine within 4 weeks prior to baseline.
- Any previous treatment with alkylating agents such as chlorambucil, or with total lymphoid irradiation.

Exclusions for general safety:

- History of severe allergic or anaphylactic reactions to human, humanised or murine monoclonal antibodies.
- Evidence of serious uncontrolled concomitant cardiovascular, nervous system, pulmonary (including obstructive pulmonary disease), renal, hepatic, endocrine (including uncontrolled diabetes mellitus) or gastrointestinal (GI) disease.
- History of diverticulitis, diverticulosis requiring antibiotic treatment, or chronic ulcerative lower GI disease such as Crohn’s disease, ulcerative colitis or other symptomatic lower GI conditions that might predispose to perforation.
- Known active current or history of recurrent bacterial, viral, fungal, mycobacterial or other infections (including but not limited to tuberculosis [TB] and atypical mycobacterial disease, hepatitis B and C, and herpes zoster, but excluding fungal infections of nail beds).
- Any major episode of infection requiring hospitalisation or treatment with IV antibiotics within 4 weeks of screening or oral antibiotics within 2 weeks of screening.
- Active TB requiring treatment within the previous 3 years. Patients should be screened for latent TB and, if positive, treated following local practice guidelines prior to initiating tocilizumab. Patients treated for TB with no recurrence in 3 years are permitted.
- Current liver disease as determined by the investigator.
- Positive hepatitis B surface antigen or hepatitis C antibody.
- Primary or secondary immunodeficiency (history of or currently active).
- Evidence of active malignant disease, malignancies diagnosed within the previous 10 years (including haematological malignancies and solid tumours, except basal and squamous cell carcinoma of the skin or carcinoma in situ of the cervix uteri that has been excised and cured), or breast cancer diagnosed within the previous 20 years.
- Pregnant women or nursing (breast-feeding) mothers.
- Patients with reproductive potential not willing to use an effective method of contraception.
- History of alcohol, drug or chemical abuse within 1 year prior to screening.
- Neuropathies or other conditions that might interfere with pain evaluation.

Laboratory exclusion criteria (at screening):

- Serum creatinine >1.4 mg/dl (124 μmol/l) in female patients and >1.6 mg/dl (141 μmol/l) in male patients.
- Alanine transaminase (ALT) or aspartate transaminase (AST) >1.5 times the upper limit of normal (ULN).
- Total bilirubin >ULN.
- Platelet count <100 x 10^9^/l (100,000/mm^3^).
- Haemoglobin <85 g/l (8.5 g/dl; 5.3 mmol/l).
- White blood cells <3.0 x 10^9^/l (3000/mm^3^).
- Absolute neutrophil count (ANC) <2.0 x 10^9^/l (2000/mm^3^).
- Absolute lymphocyte count <0.5 x 10^9^/l (500/mm^3^).

Supplementary Figure S1. Patient disposition


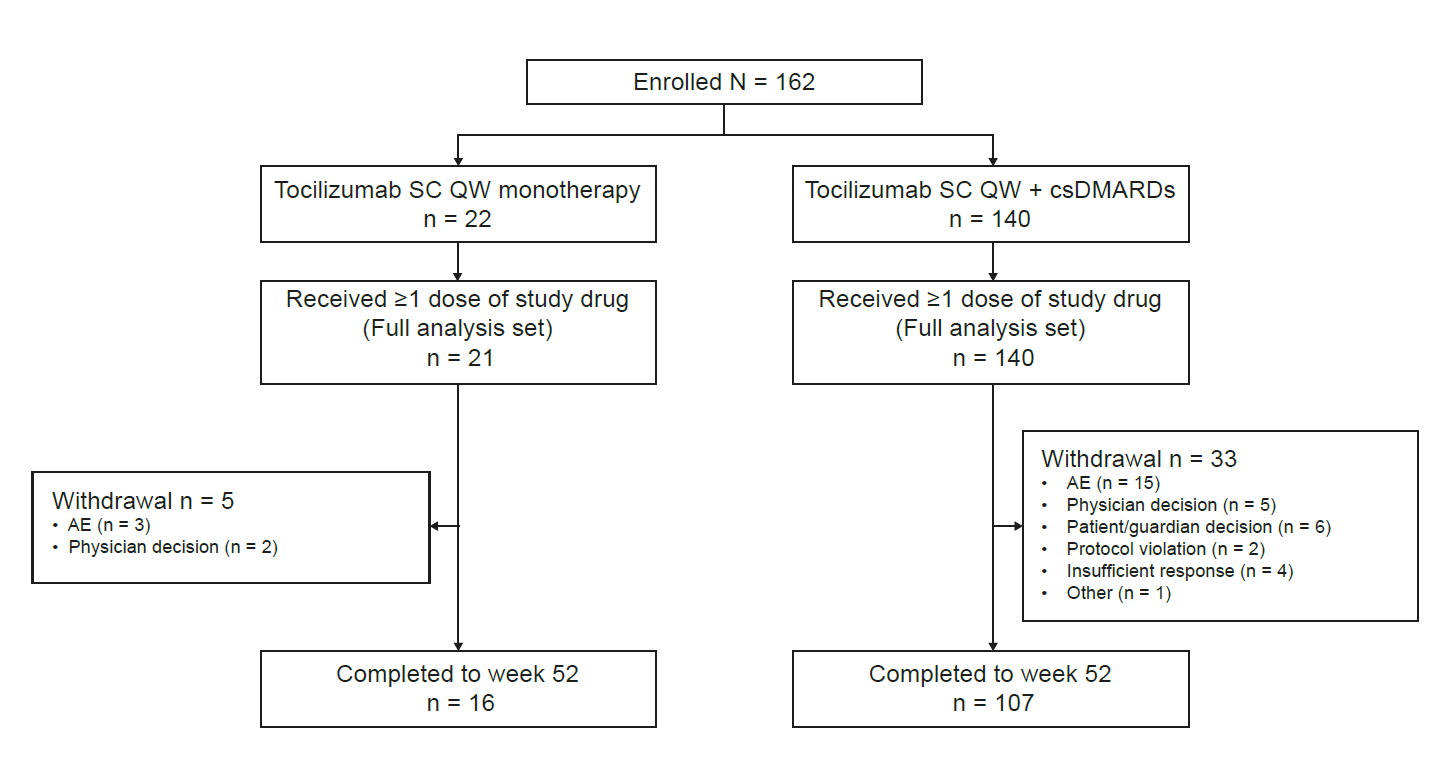
AE: adverse event; csDMARD: conventional synthetic disease-modifying anti-rheumatic drug; SC: subcutaneous; QW: once weekly.

**Supplementary Table S2.** SJC_28_ and change from baseline over time (FAS)

| SJC_28_ | TCZ-SC monotherapy (*n* = 21) | TCZ-SC + csDMARD (*n* = 140) | Total population  (*N* = 161) |
| --- | --- | --- | --- |
| **Observed value at baseline** |  |  |  |
| *n* | 21 | 139 | 160 |
| Mean (SD) | 7.0 (4.64) | 5.6 (4.43) | 5.8 (4.47) |
| **Changes from baseline** |  |  |  |
| Week 12 |  |  |  |
| *n* | 19 | 128 | 147 |
| Mean (SD) | –4.2 (4.37) | –3.8 (4.36) | –3.8 (4.35) |
| Week 24 |  |  |  |
| *n* | 19 | 117 | 136 |
| Mean (SD) | –5.9 (5.12) | –4.7 (4.21) | –4.9 (4.35) |
| Week 52 |  |  |  |
| *n* | 16 | 107 | 123 |
| Mean (SD) | –6.3 (4.81) | –5.1 (4.51) | –5.3 (4.54) |

csDMARD: conventional synthetic disease-modifying anti-rheumatic drug; FAS: full analysis set; SD: standard deviation; SJC_28_: swollen joint count on 28 joints; TCZ-SC: tocilizumab subcutaneous.

**Supplementary Table S3**. TJC_28_ and change from baseline over time (FAS)

| TJC_28_ | TCZ-SC monotherapy (*n* = 21) | TCZ-SC + csDMARD (*n* = 140) | Total population (*N* = 161) |
| --- | --- | --- | --- |
| **Observed value at baseline** |  |  |  |
| *n* | 21 | 139 | 160 |
| Mean (SD) | 10.4 (6.46) | 12.9 (7.06) | 12.6 (7.01) |
| **Changes from baseline** |  |  |  |
| Week 12 |  |  |  |
| *n* | 19 | 128 | 147 |
| Mean (SD) | –6.0 (6.71) | –8.0 (7.35) | –7.8 (7.28) |
| Week 24 |  |  |  |
| *n* | 19 | 117 | 136 |
| Mean (SD) | –8.3 (7.41) | –9.3 (7.53) | –9.1 (7.50) |
| Week 52 |  |  |  |
| *n* | 16 | 107 | 123 |
| Mean (SD) | –9.4 (7.15) | –10.4 (8.01) | –10.2 (7.89) |

csDMARD: conventional synthetic disease-modifying anti-rheumatic drug; FAS: full analysis set; SD: standard deviation; TCZ-SC: tocilizumab subcutaneous; TJC_28_: tender joint count on 28 joints.

**Supplementary Figure S2.** Mean serum concentrations of CRP from baseline to week 52 (FAS)


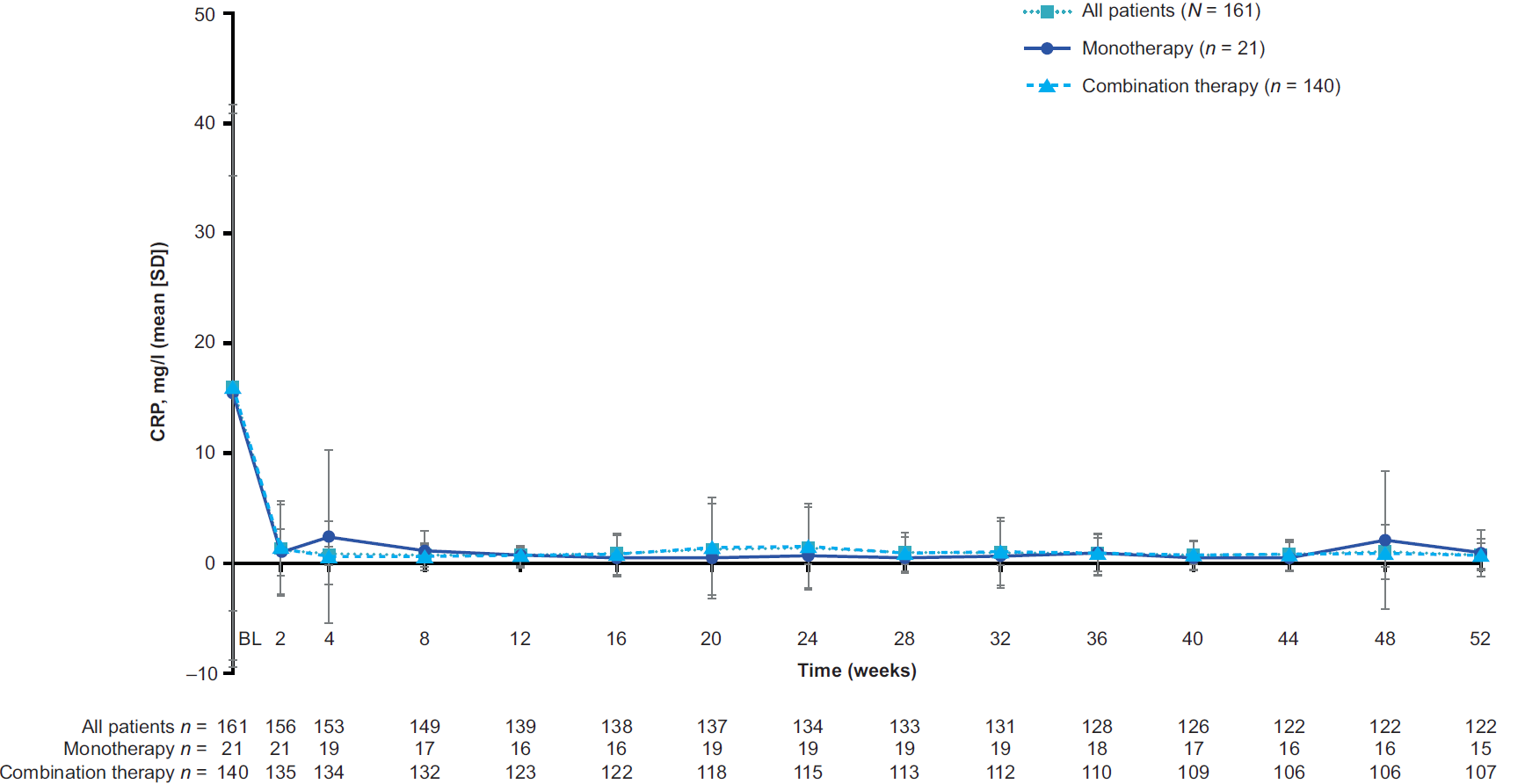


BL: baseline; CRP: C-reactive protein; FAS: full analysis set; SD: standard deviation.

**Supplementary Figure S3.** Mean ESR from baseline to week 52 (FAS)


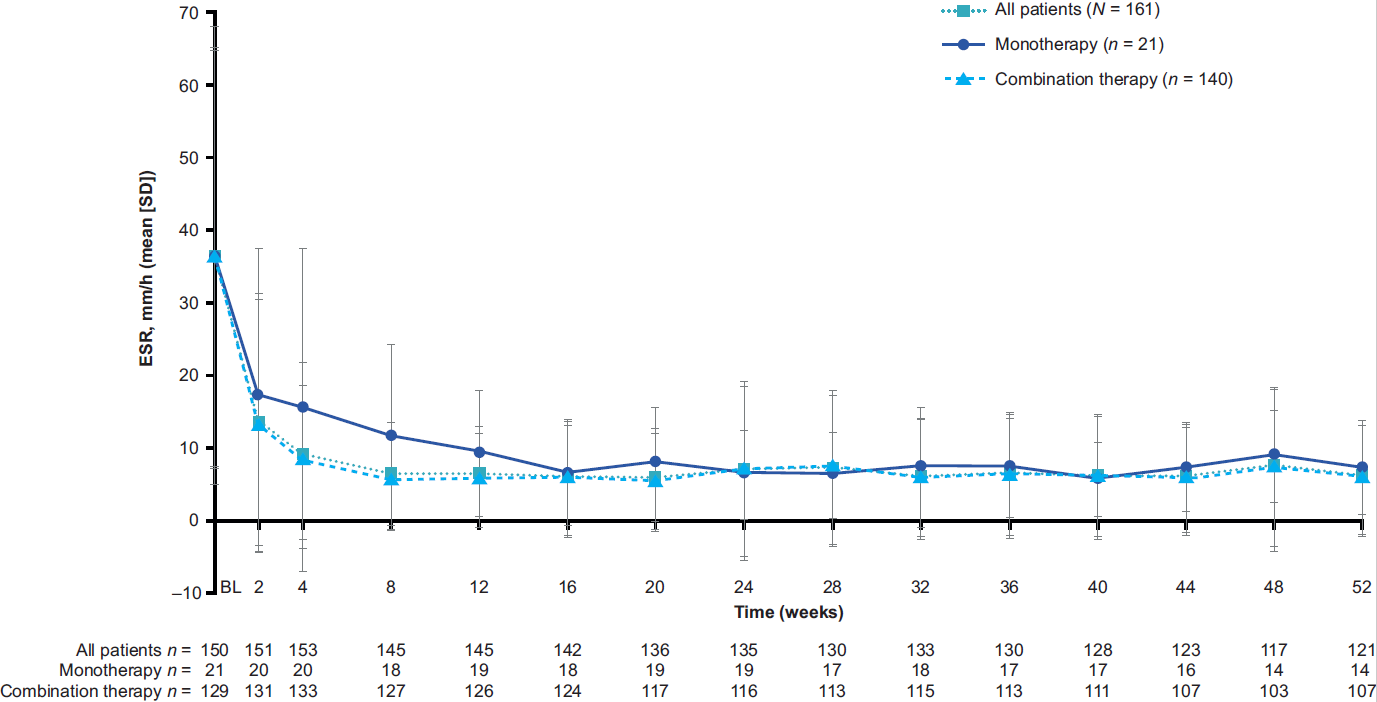


BL: baseline; ESR: erythrocyte sedimentation rate; FAS: full analysis set; SD: standard deviation.

**Supplementary Table S4.** TEAEs in ≥5% of patients in any treatment group (FAS)^a^

| *n* (%) | TCZ-SC monotherapy  (*n* = 21) | TCZ-SC + csDMARD  (*n* = 140) | Total population  (*N* = 161) |
| --- | --- | --- | --- |
| **Total number of patients with TEAEs** | **20 (95.2)** | **137 (97.9)** | **157 (97.5)** |
| Infections  Nasopharyngitis  Lower respiratory tract infection  Upper respiratory tract infection  Urinary tract infection  Oral herpes  Lower respiratory tract infection viral | 16 (76.2)  5 (23.8)  6 (28.6)  2 (9.5)  5 (23.8)  1 (4.8)  2 (9.5) | 97 (69.3)  33 (23.6)  25 (17.9)  20 (14.3)  16 (11.4)  10 (7.1)  0 | 113 (70.2)  38 (23.6)  31 (19.3)  22 (13.7)  21 (13.0)  11 (6.8)  2 (1.2) |
| Gastrointestinal disorders  Diarrhoea  Nausea  Mouth ulceration  Vomiting  Abdominal pain | 8 (38.1)  2 (9.5)  0  1 (4.8)  3 (14.3)  0 | 69 (49.3)  32 (22.9)  21 (15.0)  18 (12.9)  16 (11.4)  7 (5.0) | 77 (47.8)  34 (21.1)  21 (13.0)  19 (11.8)  19 (11.8)  7 (4.3) |
| Investigations  Alanine aminotransferase increased  Neutrophil count decreased  Blood cholesterol increased | 5 (23.8)  2 (9.5)  0  3 (14.3) | 66 (47.1)  31 (22.1)  13 (9.3)  5 (3.6) | 71 (44.1)  33 (20.5)  13 (8.1)  8 (5.0) |
| Musculoskeletal and connective tissue disorders  Arthralgia  Back pain  Pain in extremity  Rheumatoid arthritis | 8 (38.1)  1 (4.8)  3 (14.3)  0  0 | 62 (44.3)  12 (8.6)  10 (7.1)  11 (7.9)  11 (7.9) | 70 (43.5)  13 (8.1)  13 (8.1)  11 (6.8)  11 (6.8) |
| General disorders and administration site conditions  Injection site bruising  Fatigue  Injection site erythema  Influenza like illness  Peripheral swelling | 5 (23.8)  3 (14.3)  1 (4.8)  1 (4.8)  1 (4.8)  1 (4.8) | 59 (42.1)  12 (8.6)  12 (8.6)  11 (7.9)  8 (5.7)  8 (5.7) | 64 (39.8)  15 (9.3)  13 (8.1)  12 (7.5)  9 (5.6)  9 (5.6) |
| Respiratory, thoracic and mediastinal disorders  Oropharyngeal pain  Cough  Productive cough | 7 (33.3)  3 (14.3)  4 (19.0)  2 (9.5) | 57 (40.7)  27 (19.3)  21 (15.0)  3 (2.1) | 64 (39.8)  30 (18.6)  25 (15.5)  5 (3.1) |
| Skin and subcutaneous tissue disorders  Rash | 4 (19.0)  0 | 55 (39.3)  18 (12.9) | 59 (36.6)  18 (11.2) |
| Injury, poisoning and procedural complications  Contusion  Fall  Laceration | 5 (23.8)  2 (9.5)  1 (4.8)  0 | 43 (30.7)  13 (9.3)  14 (10.0)  7 (5.0) | 48 (29.8)  15 (9.3)  15 (9.3)  7 (4.3) |
| Nervous system disorders  Headache  Dizziness  Migraine  Paraesthesia  Lethargy | 9 (42.9)  4 (19.0)  2 (9.5)  1 (4.8)  2 (9.5)  0 | 36 (25.7)  13 (9.3)  8 (5.7)  7 (5.0)  6 (4.3)  7 (5.0) | 45 (28.0)  17 (10.6)  10 (6.2)  8 (5.0)  8 (5.0)  7 (4.3) |
| Blood and lymphatic system disorders  Neutropenia | 0  0 | 20 (14.3)  14 (10.0) | 20 (12.4)  14 (8.7) |
| Eye disorders | 2 (9.5) | 13 (9.3) | 15 (9.3) |
| Reproductive system and breast disorders | 1 (4.8) | 11 (7.9) | 12 (7.5) |
| Vascular disorders | 0 | 12 (8.6) | 12 (7.5) |
| Metabolism and nutrition disorders | 1 (4.8) | 9 (6.4) | 10 (6.2) |
| Ear and labyrinth disorders | 0 | 8 (5.7) | 8 (5.0) |
| Immune system disorders | 0 | 8 (5.7) | 8 (5.0) |
| Psychiatric disorders | 3 (14.3) | 5 (3.6) | 8 (5.0) |
| Surgical and medical procedures | 3 (14.3) | 3 (2.1) | 6 (3.7) |

^a^If a patient experienced more than one TEAE, the patient was counted once for each system organ class and once for each preferred term.

csDMARD: conventional synthetic disease-modifying anti-rheumatic drug; FAS: full analysis set; TCZ-SC: tocilizumab subcutaneous; TEAE: treatment-emergent adverse event.

**Supplementary Table S5.** TESAEs occurring in any treatment group (FAS)^a^

| *n* (%) | TCZ-SC monotherapy  (*n* = 21) | TCZ-SC + csDMARD  (*n* = 140) | Total population  (*N* = 161) |
| --- | --- | --- | --- |
| **Total number of patients with TESAEs** | **3 (14.3)** | **7 (5.0)** | **10 (6.2)** |
| Infections and infestations  Arthritis bacteria  Cellulitis  Pneumonia  Sinusitis  Subcutaneous abscess | 1 (4.8)  0  0  1 (4.8)^b^  1 (4.8)^b^  0 | 3 (2.1)  1 (0.7)  1 (0.7)  0  0  1 (0.7)^b^ | 4 (2.5)  1 (0.6)  1 (0.6)  1 (0.6)  1 (0.6)  1 (0.6) |
| Injury, poisoning and procedural complications  Accidental overdose  Wound | 1 (4.8)  0  1 (4.8) | 1 (0.7)  1 (0.7)  0 | 2 (1.2)  1 (0.6)  1 (0.6) |
| Cardiac disorders  Atrial fibrillation | 0  0 | 1 (0.7)  1 (0.7) | 1 (0.6)  1 (0.6) |
| Gastrointestinal disorders  Vomiting | 1 (4.8)  1 (4.8) | 0  0 | 1 (0.6)  1 (0.6) |
| Musculoskeletal and connective tissue disorders  Costochondritis | 0  0 | 1 (0.7)  1 (0.7) | 1 (0.6)  1 (0.6) |
| Neoplasms benign, malignant and unspecified (including cysts and polyps)  Prostate cancer | 1 (4.8)  1 (4.8) | 0  0 | 1 (0.6)  1 (0.6) |
| Respiratory, thoracic and mediastinal disorders  Pulmonary fibrosis | 0  0 | 1 (0.7)  1 (0.7)^b^ | 1 (0.6)  1 (0.6) |
| Skin and subcutaneous tissue disorders  Drug eruption | 0  0 | 1 (0.7)  1 (0.7)^b^ | 1 (0.6)  1 (0.6) |
| Surgical and medical procedures  Knee arthroplasty | 1 (4.8)  1 (4.8) | 0  0 | 1 (0.6)  1 (0.6) |

^a^If a patient experienced more than one TESAE, the patient was counted once for each system organ class and once for each preferred term.

^b^Four patients experienced five TESAEs that were considered related to study medication: pneumonia, sinusitis, subcutaneous abscess, pulmonary fibrosis and drug eruption.

csDMARD: conventional synthetic disease-modifying anti-rheumatic drug; FAS: full analysis set; TCZ-SC: tocilizumab subcutaneous; TESAE: treatment-emergent serious adverse event.

**Supplementary Table S6.** Individual TEAEs of special interest occurring in ≥3% of patients in any treatment group (FAS)^a^

| *n* (%) | TCZ-SC monotherapy  (*n* = 21) | TCZ-SC + csDMARD  (*n* = 140) | Total population  (*N* = 161) |
| --- | --- | --- | --- |
| **Total number of patients with TEAEs of special interest** | **9 (42.9)** | **61 (43.6)** | **70 (43.5)** |
| Skin and subcutaneous tissue disorders  Rash  Rash macular  Petechiae  Dermatitis allergic | 2 (9.5)  0  1 (4.8)  1 (4.8)  1 (4.8) | 31 (22.1)  18 (12.9)  0  1 (0.7)  0 | 33 (20.5)  18 (11.2)  0  2 (1.2)  1 (0.6) |
| General disorders and administration site conditions  Injection site bruising | 3 (14.3)  3 (14.3) | 16 (11.4)  12 (8.6) | 19 (11.8)  15 (9.3) |
| Injury, poisoning and procedural complications  Contusion | 2 (9.5)  2 (9.5) | 13 (9.3)  13 (9.3) | 15 (9.3)  15 (9.3) |
| Respiratory, thoracic and mediastinal disorders  Epistaxis | 1 (4.8)  1 (4.8) | 7 (5.0)  4 (2.9) | 8 (5.0)  5 (3.1) |
| Reproductive system and breast disorders  Vaginal haemorrhage | 1 (4.8)  1 (4.8) | 3 (2.1)  2 (1.4) | 4 (2.5)  3 (1.9) |
| Neoplasms benign, malignant and unspecified (including cysts and polyps)  Prostate cancer | 1 (4.8)  1 (4.8) | 2 (1.4)  0 | 3 (1.9)  1 (0.6) |

^a^TEAEs of special interest were identified via SMQ.

csDMARD: conventional synthetic disease-modifying anti-rheumatic drug; FAS: full analysis set; SMQ: standardised Medical Dictionary for Regulatory Activities (MedDRA) query (SMQ); TCZ-SC: tocilizumab subcutaneous; TEAE: treatment-emergent adverse event.
